# Variation in mycorrhizal growth response among a spring wheat mapping population shows potential to breed for symbiotic benefit

**DOI:** 10.1002/fes3.370

**Published:** 2022-02-14

**Authors:** Tom J. Thirkell, Mike Grimmer, Lucy James, Daria Pastok, Théa Allary, Ashleigh Elliott, Neil Paveley, Tim Daniell, Katie J. Field

**Affiliations:** ^1^ Plants, Photosynthesis and Soil School of Biosciences University of Sheffield Sheffield UK; ^2^ ADAS Boxworth Cambridge UK; ^3^ School of Biology Centre for Plant Sciences University of Leeds Leeds UK; ^4^ ADAS High Mowthorpe Duggleby UK; ^5^ Present address: Fera Science Ltd Sand Hutton UK

**Keywords:** arbuscular mycorrhiza, fungi, mycorrhizal growth response, phosphorus, sustainable agriculture, *Triticum aestivum* (wheat)

## Abstract

All cereal crops engage in arbuscular mycorrhizal symbioses which can have profound, but sometimes deleterious, effects on plant nutrient acquisition and growth. The mechanisms underlying variable mycorrhizal responsiveness in cereals are not well characterised or understood. Adapting crops to realise mycorrhizal benefits could reduce fertiliser requirements and improve crop nutrition where fertiliser is unavailable. We conducted a phenotype screen in wheat (*Triticum aestivum* L.), using 99 lines of an Avalon × Cadenza doubled‐haploid mapping population. Plants were grown with or without a mixed inoculum containing 5 species of arbuscular mycorrhizal fungi. Plant growth, nutrition and mycorrhizal colonisation were quantified. Plant growth response to inoculation was remarkably varied among lines, ranging from more than 30% decrease to 80% increase in shoot biomass. Mycorrhizal plants did not suffer decreasing shoot phosphorus concentration with increasing biomass as observed in their non‐mycorrhizal counterparts. The extent to which mycorrhizal inoculation was beneficial for individual lines was negatively correlated with shoot biomass in the non‐mycorrhizal state but was not correlated with the extent of mycorrhizal colonisation of roots. Highly variable mycorrhizal responsiveness among closely related wheat lines and the identification of several QTL for these traits suggests the potential to breed for improved crop‐mycorrhizal symbiosis.

## 1 INTRODUCTION

The domestication and improvement of a small number of cereal species has given rise to the staple crops which now underpin much of human nutrition. In wheat, yields have increased dramatically since the 1950s with fertiliser and pesticide applications, and the inclusion of several key genes into elite cultivars, to confer, for example, a semi‐dwarfing growth habit, disease resistance and regulation of flowering (Morrell et al., 2012; Pingali, 2012). Despite these advances, nutrient acquisition still limits grain production in many systems (Kvakić et al., 2018; Vitousek et al., 2009). In many countries, cereal yields in intensive arable systems are no longer increasing (Grassini et al., 2013). In high‐input systems, phosphorus (P) limitation in cereal crops is particularly concerning, as P fertiliser production relies largely on the supply of rock phosphate, a finite resource (Cordell & White, 2014).

In highly productive arable systems, P limitation arises because most fertiliser P becomes immobilised in soil in forms which plants are unable to acquire (Tinker & Nye, 2000). Regular applications of P mean that much of the arable land in the developed world has a stock of ‘legacy P’ (Rowe et al., 2016). Although these soils are heavily enriched in P, low phytoavailability means many cereals remain P‐limited (YEN, 2021). In high‐input systems, elevated soil P concentrations can lead to increased leaching and run‐off, with ecologically damaging consequences downstream (Elser & Bennett, 2011; Withers & Haygarth, 2007). Mounting evidence suggests that by exploiting legacy P reserves, cereal yields could be maintained, P fertiliser applications reduced, and the severity of environmental harm diminished (Rowe et al., 2016; Withers et al., 2017). Exploiting legacy P will likely require crops with greater P acquisition efficiency. In soils where there is little legacy P, higher crop P acquisition efficiency should allow P fertiliser applications to be reduced towards levels closer to those which are removed from the field as grain.

Exploiting soil microbes could provide a means by which P acquisition efficiency from arable soils may be increased. Cereal crop species worldwide engage in symbioses with arbuscular mycorrhizal (AM) fungi (Smith & Smith, 2011), a group of soil‐dwelling fungi in the clade Glomeromycotina (Spatafora et al., 2016). The symbiosis is characterised by the development of specialised fungal structures within the cortex of host plant roots, facilitating the transfer of mineral nutrients from fungus to plant. For the host plant, the principal benefit of the symbiosis is most commonly enhanced P uptake (Smith & Read, 2008). With a dense network of hyphae proliferating into the soil, a mycorrhizal plant may acquire P from a much greater volume of soil than a non‐mycorrhizal plant. In return for mineral nutrients acquired from the soil, plant carbon (C) is acquired by the fungus in the form of sugars and lipids (Luginbuehl et al., 2017). In addition to nutritional advantages, AM fungi may also improve plant water use efficiency, heavy metal tolerance and ability to withstand attack from pests and pathogens (Cameron et al., 2013). Beyond the host plant, soils with greater quantities of AM fungal biomass may be more stable against erosion, suffer less water and nutrient leaching and potentially have greater carbon storage capacity (Rillig et al., 2019).

Although frequently identified as a mechanism which may aid sustainable intensification in agriculture (Rillig et al., 2016; Sosa‐Hernández et al., 2019; Thirkell et al., 2017), the utilisation of arbuscular mycorrhizas in cropping systems is limited by the unreliable nature of the interaction, in terms of tangible, realised plant benefits (Rillig et al., 2019; Ryan et al., 2019). Highly varied and unpredictable nutritional and yield responses among mycorrhizal plants have been demonstrated in numerous crop species including wheat (Hetrick et al., 1993; Lehnert et al., 2018; Singh et al., 2012; Zhu et al., 2001). Frequently observed yield reductions in mycorrhizal crop plants compared with non‐mycorrhizal counterparts have led to a persistent stance in the literature that AM fungi are likely to be of little benefit in conventional, intensive agricultural systems (Ryan & Graham, 2018). However, recent meta‐analyses suggest overall positive outcomes for grain yield following mycorrhizal colonisation or inoculation (Lehmann et al., 2012; Zhang et al., 2019), and increasing adoption of sustainable practices (Rillig et al., 2019; Ryan et al., 2019) suggests that application of mycorrhizas in agriculture warrants further attention. This is especially pertinent for those farming practices which more actively prioritise soil ecology and environmental impacts, such as organic or regenerative agricultural systems (LaCanne & Lundgren, 2018; Reganold & Wachter, 2016).

As far as we are aware, deliberate selection for positive (or negative) mycorrhizal traits has never occurred in the development of modern elite cereal cultivars since domestication of progenitors c. 8000 years ago. Crop breeding may have inadvertently selected against mutualistic mycorrhizal associations, as plant traits become adapted for roles which were carried out by fungal symbionts in ancestral progenitors. Breeding for greater root length densities in upper soil horizons, for example, can improve plant P acquisition (White et al., 2013), but may make AM symbioses less important, as fine roots substitute for AM fungal mycelia in acquiring P (Raven et al., 2018). As crops are further bred for nutrient acquisition under high rates of fertiliser application, the redundancy of mycorrhizal symbiosis is likely to be increased. Where plants are unable to dissociate from their fungal symbionts, plant carbon is acquired by the fungi for reduced mineral nutrient cost, and the symbiosis may no longer be mutualistic. Progress in crop breeding to reduce susceptibility to fungal disease such as take‐all (McMillan et al., 2014) may unintentionally also reduce susceptibility to colonisation by beneficial symbionts such as AM fungi, further exacerbating this problem (Jacott et al., 2017).

Unknowingly including or excluding mycorrhizal traits is potentially very significant, given the fact that AM fungi can, in extreme cases, be responsible for all plant P uptake and may acquire more than 10% of plant C. For some time, it has been suggested that cereal crops might be bred to exploit their symbiosis with AM fungi (Berger & Gutjahr, 2021; Kaeppler et al., 2000; Lefebvre, 2020; Sawers et al., 2008). Wheat (*Triticum aestivum* L.) is grown on more land than any other crop, is ubiquitously mycorrhizal and shows varied nutritional and growth responses to AM fungal colonisation (Hetrick et al., 1992, 1993; Lehnert et al., 2018). A number of genetic markers in wheat appear to be associated with the degree to which wheat genotypes become colonised by AM fungi (De Vita et al., 2018; Lehnert et al., 2017). Improved mycorrhizal responsiveness to colonisation is probably a better target for breeders than purely seeking to increase the biomass of mycorrhizal fungi within host roots, as the extent of colonisation is not necessarily correlated with the benefit afforded to host plants, in nutrient assimilation or biomass (Martin et al., 2012; Sawers et al., 2017; Smith et al., 2004). Here, we used a greenhouse phenotype screen to characterise the variation in growth and nutritional responses of a panel of 99 spring wheat lines from a doubled‐haploid mapping population (progeny from a cross of cv. Avalon × cv. Cadenza) to inoculation with a mixed community of five AM fungal species. Composite interval mapping was then used to identify quantitative trait loci (QTL) associated with mycorrhizal benefit in the population.

## 2 MATERIALS AND METHODS

### 2.1 Wheat and mycorrhizal fungal material

A subset of 99 spring wheat (*Triticum aestivum* L.) lines were selected from the Avalon × Cadenza doubled‐haploid mapping population (Table S1), the UK reference population which represents a wide range of the observable variation in UK elite wheat germplasm, including contrasting mycorrhizal phenotypes (Elliott et al., 2021). The population of doubled‐haploid (DH) individuals, derived from F1 progeny of a cross between cultivars Avalon and Cadenza, was developed at the John Innes Centre, as part of a DEFRA (Department of Environment, Food and Rural Affairs, UK Government) project led by ADAS.

Seeds were surface sterilised (1% sodium hypochlorite solution, 5 min) then rinsed thoroughly in distilled H_2_O, before being planted singly into ‘Jumbo Rootrainer’ pots measuring 6.32 × 6.32 × 25 cm (WxDxH) (Tildenet, Bristol, UK), filled with a 50/50 (v/v) mix of perlite and silica sand. Each pot in the mycorrhizal treatment received 10 g of wetted (with autoclaved, distilled H_2_O) inoculum (Rootgrow Professional^®^; PlantWorks Ltd, Sittingbourne, UK). This inoculum contained the AM fungal species *Funneliformis mosseae*, *Funneliformis geosporus*, *Claroideoglomus clarodeum*, *Rhizophagus intraradices* and *Glomus microaggregatum* and comprised small root fragments, AM fungal spores and a granulated clay. To the non‐mycorrhizal treatment, each pot received 10 g of a twice autoclaved (121°C for 20 min, 48 h between cycles) portion of the same inoculum. Inoculum was autoclaved twice to reduce the risk of contamination in the non‐mycorrhizal treatment by AM fungal spores which may have survived the first autoclave cycle. In both treatments, inoculum was added as a layer at the bottom of the planting hole to which the seedling was added, to ensure root growth through inoculum and thereby maximise chances of AM fungal colonisation.

Five replicate plants of each line were grown in each of the mycorrhizal and non‐mycorrhizal treatments, such that 990 plants were grown in total. Planting was carried out in 5 blocks, each separated by one week; each block contained one mycorrhizal and one non‐mycorrhizal replicate per line. Within blocks, lines were spatially randomised, while mycorrhizal and non‐mycorrhizal counterparts of each line were placed adjacent, to minimise environmental artefacts on seedling growth. The first block was planted 11–12 July 2018, and the last block planted 8–9 August 2018 (see Table S2 for full planting and harvesting timings). Plants were maintained in a heated, lit glasshouse (16‐hour day length, day temperature: 22°C, night temperature: 17°C). Supplementary lighting provided 202.9 ± 12.1 µmol m^−2^ s^−1^ at canopy height. Relative humidity was maintained at 70% for the duration of the growing period.

From two weeks after planting, each pot received weekly 30 ml doses of Long Ashton nutrient solution prepared to the ‘nitrate type’ protocol, modified by reducing the monosodium phosphate component to 25% of the original protocol (see Table S3). Plants were watered with tap water as required through the course of the experiment. Where plants did not grow, these individual replicates were excluded from analyses (data shown in Table S4).

### 2.2 Plant harvest and sample preparation

At 5 weeks (immediately prior to harvest), shoot height was measured from the soil surface to the tip of the tallest leaf. Plants were destructively harvested at 5 weeks (block 1: 15–17 August, block 5: 12–14 September; see Table S2 for full planting and harvest timings). Plants were removed from pots, and roots were gently washed from the growth medium. Shoot and root material were separated. After patting dry with tissue paper, root fresh biomass was recorded, and a small subsample (c. 10%–20% root system) was taken and stored in 50% (v/v) ethanol to allow subsequent quantification of mycorrhizal colonisation. The remaining root fresh biomass was recorded. Shoot and root samples were oven‐dried at 70°C for 60 h and dry biomasses recorded. Total root dry biomass was calculated by extrapolating from total fresh biomass, fresh biomass of remaining sample and dry biomass of remaining sample.

### 2.3 Tissue phosphorus measurement

Shoot phosphorus (P) content and concentration were quantified in mycorrhizal and non‐mycorrhizal counterparts from 50 lines (randomly selected, see Table S5) from block 1. Dried shoot material was homogenised (IKA A10 basic mill; IKA & Co, Staufen, Germany), then samples of known mass (25–50 mg) were digested in 1 ml H_2_SO_4_ (96% v/v) at 360°C for 15 min (BTD5 dry block heater; Grant Instruments, Shepreth, UK). Digest products were allowed to cool to 20°C before addition of 100 µl H_2_O_2_ (30% v/v), at which point samples became colourless. Sample P content was determined by colorimetric methods adapted from Murphy and Riley (1962) and used in Thirkell et al. (2020). Briefly, 0.2 ml aliquots of digest samples were mixed with 0.2 ml l‐ascorbic acid, 0.2 ml 3.44 M NaOH and 0.5 ml of developer solution (prepared by dissolving 0.1 g antimony potassium tartrate and 4.8 g ammonium molybdate in 250 ml 2 M H_2_SO_4_). After incubating at 25°C for 45 min, absorbance was read at 882 nm with a Jenway 6300 spectrophotometer (Cole‐Palmer: St Neots, UK). Using a calibration curve produced with a 10 ppm P standard solution (NaH_2_PO_4_), digest sample P concentrations were determined.

### 2.4 Assessment of mycorrhizal colonisation

Root colonisation by AM fungi was confirmed for all plants in the mycorrhizal group, and AM fungal absence was confirmed in all plants in the non‐mycorrhizal group. Using methods adapted from Vierheilig et al. (1998), root subsamples were cleared in 10% (w/v) KOH for 40 min at 70°C, briefly rinsed in tap water, immersed in staining solution (5% Pelikan ‘Brilliant Black’ ink; Pelikan Holding AG, Hanover, Germany, 5% acetic acid, 90% distilled H_2_O) for 20 min at 20°C and then incubated for 48 h at 20°C in 1% acetic acid. For each plant, 15 sections of root (of length c. 1 cm) were mounted to microscope slides with PVLG (8.33 g polyvinyl alcohol, 50 ml distilled H_2_O, 50 ml lactic acid) and fixed in a drying oven at 65°C for 24 h. AM fungal colonisation was quantified for all plants in the mycorrhizal treatment of 36 lines from the population, categorised as having positive/neutral/negative shoot biomass response to inoculation (Table S6). Mycorrhizal colonisation was quantified using the methods of McGonigle et al. (1990), following inspection of a minimum of 100 intersects per plant.

### 2.5 Data handling and analysis

The effect of inoculation on wheat shoot dry biomass (hereafter mycorrhizal growth response, MGR) was calculated following Hetrick et al. (1992), using the formula MGR = (mycorrhizal shoot mass ‐ mean non‐mycorrhizal shoot mass)/mean non‐mycorrhizal shoot mass. Calculating MGR separately for each replicate within the mycorrhizal group, while comparing against the mean value in the non‐mycorrhizal group allowed 5 replicate values to be generated for each line. Mycorrhizal response values were also computed for root dry biomass, plant dry biomass, root weight ratio (the proportion of the plant dry biomass that is root dry biomass, calculated as root dry biomass/plant dry biomass) and shoot height. Shoot phosphorus response to inoculation (MPR) was calculated similarly.

All statistical analyses of phenotype traits were performed using the RStudio interface of R statistical software, version 3.4.3. (R Core Team, 2020; RStudio Team, 2015). Wilcoxon sum rank tests were performed to test differences between mycorrhizal vs non‐mycorrhizal trait means where contrasts are tested by AM treatment across the whole population, for example comparing shoot biomass between inoculated and uninoculated plants. To test where MGR values were significantly different from zero, one‐sample Wilcoxon signed‐rank tests were performed. Spearman rank correlation was used to test relationships between continuous variables; shoot biomass vs shoot phosphorus concentration, and root length colonisation vs shoot phosphorus concentration. Kruskal–Wallis rank‐sum tests were performed on population data to determine whether line identity significantly affected MGR traits.

### 2.6 QTL identification

Linkage maps and molecular marker data for the Avalon x Cadenza mapping population were obtained from the University of Bristol Cereals DB website (cerealsdb.uk.net/cerealgenomics/). Linkage groups were tested for the presence of segregating quantitative trait loci (QTL) using the composite interval mapping (CIM; Zeng, 1994) function of Windows QTL Cartographer version 2.5 software (Wang, Basten, & Zeng, 2012; Wang, Schornack, et al., 2012). Automatic cofactor selection by a forward regression was performed using 5 control markers and a window size of 10 cM, under the standard CIM model. The step size chosen for all traits was 1 cM. QTL were deemed significant above a LOD value of 3.0.

## 3 RESULTS

### 3.1 Mycorrhizal inoculation elicits variable growth responses

**FIGURE 1:**
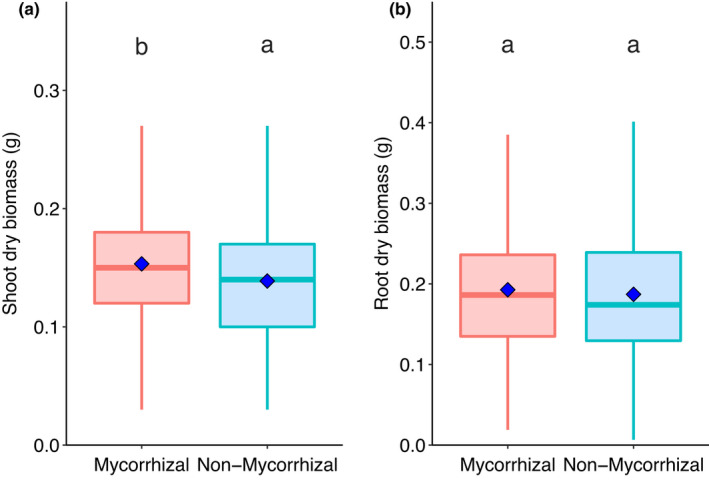
Comparison of (a) shoot dry biomass and (b) root dry biomass in mycorrhiza‐inoculated and non‐mycorrhizal wheat (*Triticum aestivum* L.) plants. Boxes sharing letters are not significantly different, as determined by Wilcoxon signed‐rank test. Blue diamonds represent mean values for boxplot data. All replicate plants of 99 lines of Avalon × Cadenza DH mapping population are represented, *n* = 445

Considering all lines from the Avalon × Cadenza mapping population together, inoculation with AM fungi increased shoot dry biomass by over 10% (*W* = 125816, *p* < 0.0001; Figure 1a), while root biomass did not differ between AM‐inoculated and mock‐inoculated groups (*W* = 112344, *p* = 0.25; Figure 1b). Mycorrhizal inoculation increased shoot biomass sufficiently to increase total dry biomass of wheat plants (*W* = 117774, *p* = 0.013; Figure S1a). Root weight ratio (the proportion of plant biomass that is root) was significantly reduced in mycorrhizal plants compared with non‐mycorrhizal counterparts, as inoculation increased shoot biomass while root biomass was unchanged (*W* = 94574, *p* = 0.001; Figure S1b). Shoot height was also significantly increased in mycorrhizal compared with non‐mycorrhizal plants (*W* = 122680, *p* < 0.001; Figure S1c).

**FIGURE 2:**
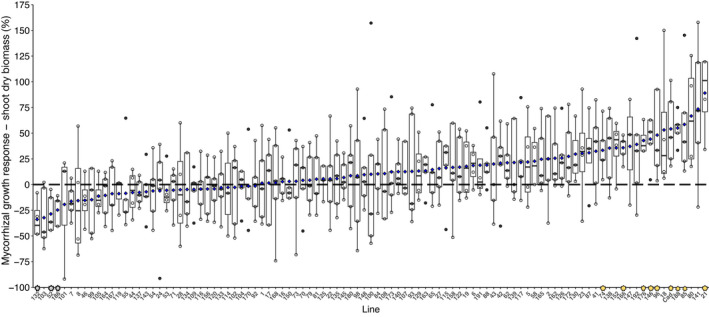
Response of wheat (*Triticum aestivum* L.) shoot dry biomass to arbuscular mycorrhizal inoculation. Boxes represent individual wheat lines from the Avalon × Cadenza DH mapping population. Boxes are ranked by mean response to inoculation. Blue diamonds on boxes represent mean MGR value for that line. Grey stars on the *x*‐axis denote lines where Wilcoxon signal rank test shows the mean MGR is significantly lower than zero; yellow stars show lines where the mean value is significantly higher than zero. Except where noted in Table S3, *n* = 5

Substantial variation in trait response to mycorrhizal inoculation was found among the 99 lines tested (Tables S7 and S8). Shoot dry biomass response to inoculation varied from −34% to +89% among individual lines (Kruskal–Wallis: *χ* ^2^ = 136.86, df = 98, *p* = 0.0058; Figure 2). Wilcoxon signed‐rank tests indicated that 3 lines showed statistically significant negative MGR, and 9 lines showed statistically significant positive shoot MGR (marked on the *x*‐axis in Figure 2 with grey and yellow stars, respectively. Tables S6 and S7). Similar trait variation was shown for root biomass (*χ* ^2^ = 158.64, df = 98, *p* = 0.0001), total biomass (*χ* ^2^ = 157.19, df = 98, *p* = 0.0001), root weight ratio (*χ* ^2^ = 143.78, df = 98, *p* = 0.0018) and shoot height (*χ* ^2^ = 177.61, df = 98, *p* < 0.0001) (Figures S2–S5). Following mycorrhizal inoculation, root biomass was significantly increased in 4 lines and decreased in 8 lines (Figure S2, Tables S7 and S8). Total dry biomass was increased in 5 lines and decreased in 6 lines (Figure S3, Tables S7 and S8). Root weight ratio response to inoculation exhibited the lowest variability of the traits measured here, ranging from −21% to +22% among lines, although was significantly increased in 3 lines and decreased in 8 lines (Figure S4, Tables S7 and S8). Shoot height response to inoculation also showed significant variation among lines (Figure S5), ranging from −25% to +33%. Inoculation was far more likely to increase shoot height than to decrease it, being statistically significantly different from zero in 14 lines and 1 line, respectively (Figure S5, Tables S7 and S8).

**FIGURE 3:**
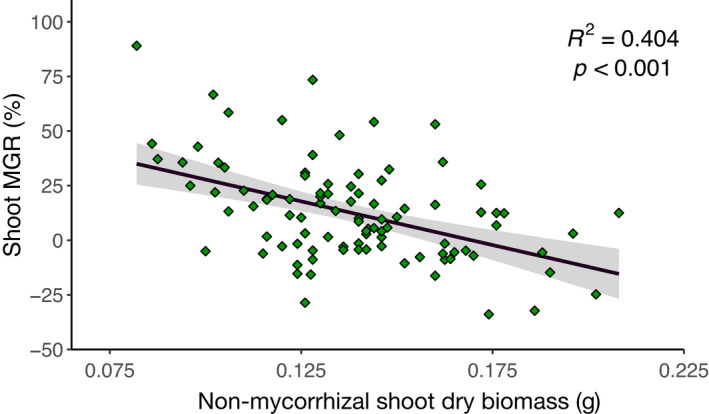
Association between mean mycorrhizal growth response of 99 lines of Avalon × Cadenza DH wheat (*Triticum aestivum* L.) mapping population and shoot dry biomass of non‐mycorrhizal replicates. Negative correlation (Spearman rank) indicates that mycorrhizal responsiveness is most positive in those lines which have a lower shoot dry biomass in the non‐mycorrhizal state; lines with a high dry biomass when non‐mycorrhizal are more likely to experience a negative MGR. Data points are mean values for traits of each line. Except where noted in Table S3, *n* = 5

Mean shoot biomass among lines was significantly negatively correlated with mycorrhizal growth response (Figure 3); those lines with the highest biomass in the non‐mycorrhizal treatments were more likely to exhibit negative mycorrhizal growth responses, while the lines with the lowest non‐mycorrhizal biomass were more likely to have positive mycorrhizal growth responses.

### 3.2 Shoot phosphorus uptake

**FIGURE 4:**
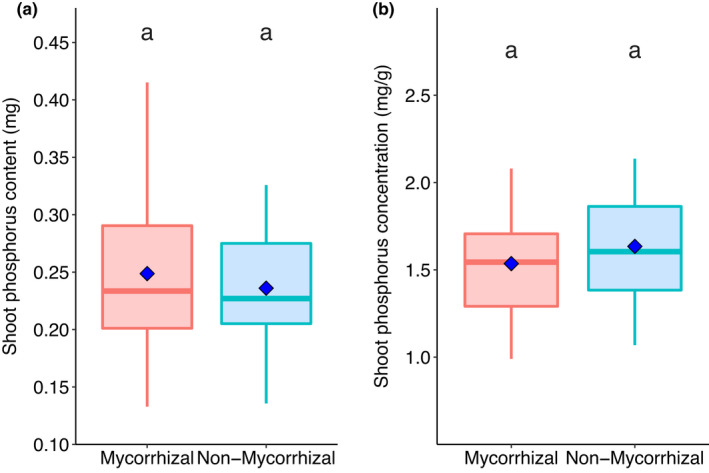
Comparison of (a) shoot phosphorus content and (b) shoot phosphorus concentration in mycorrhiza‐inoculated and non‐mycorrhizal wheat (*Triticum aestivum* L.) plants. Boxes sharing letters are not significantly different, as determined by Wilcoxon signed‐rank test. Blue diamonds represent mean values for boxplot data. *n* = 50

**FIGURE 5:**
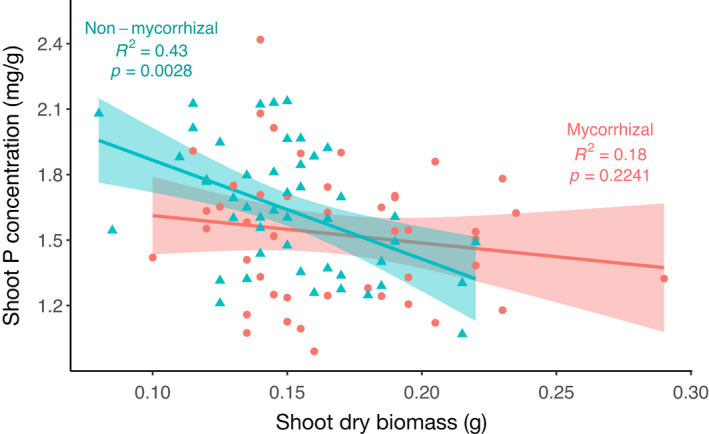
Scatterplot and Spearman rank correlation of wheat (*Triticum aestivum* L.) shoot dry biomass and shoot phosphorus concentration, with mycorrhizal‐inoculated and mock‐inoculated plotted separately in pink and blue, respectively. Single representative replicates from 50 lines of Avalon × Cadenza DH mapping population are represented, *n* = 50

Overall, AM inoculation did not increase P content or concentration of shoots (*p* > 0.05; Figure 4a,b). Plotting P data against shoot dry biomass, however, revealed effects of AM inoculation on P concentration (Figure 5). Pearson rank correlation showed that shoot P concentration is significantly negatively correlated with shoot dry biomass in non‐mycorrhizal plants (*R* ^2^ = −0.43, *p* = 0.0028), suggesting that non‐mycorrhizal plants suffer a relative dilution in shoot P with increased biomass (Figure 5). By contrast, there was no correlation between shoot biomass and P concentration in the mycorrhizal‐inoculated plants (*R* ^2^ = −0.18, *p* = 0.2241), suggesting that larger plants in the mycorrhizal treatment were better able to maintain shoot P concentration than those in the non‐mycorrhizal group. Similarly, root dry biomass was negatively correlated with shoot P concentration in non‐mycorrhizal, but not mycorrhizal plants (Figure S6), indicating more efficient P uptake and assimilation in mycorrhizal plants compared with non‐mycorrhizal counterparts.

### 3.3 Arbuscular mycorrhizal colonisation

Root length colonisation data were collected from lines representative of positive, neutral and negative shoot biomass responses (Table S6). All plants in the mycorrhizal treatment were colonised by arbuscular mycorrhizal hyphae, with many also containing characteristic arbuscules and vesicles. All plants in the non‐mycorrhizal control group remained free from AM fungal colonisation. A Kruskal–Wallis rank‐sum test showed there was no difference in the per cent root length colonisation between groups categorised as having negative, neutral or positive shoot MGR (*χ* ^2^ = 4.19, df = 2, *p* = 0.123; Figure S7, Table S6). Similarly, there was no difference between MGR groups in terms of the frequency of arbuscules (*χ* ^2^ = 0.413, df = 2, *p* = 0.813) or vesicles (*χ* ^2^ = 1.47, df = 2, *p* = 0.479).

**FIGURE 6:**
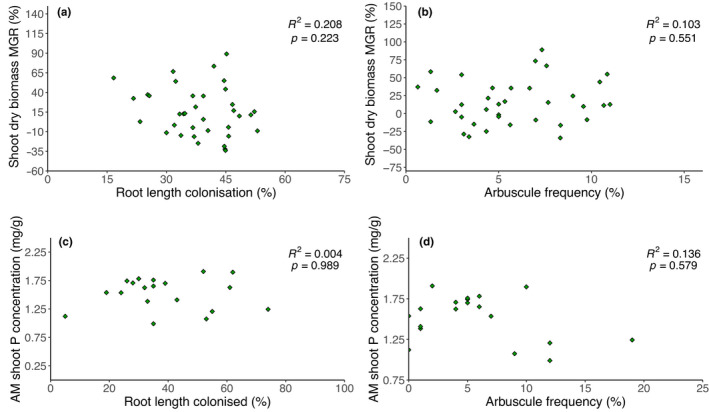
Scatterplots of mycorrhizal growth response (shoot dry biomass) plotted against (a) extent of root colonisation by arbuscular mycorrhizal fungi and (b) frequency of arbuscules; and shoot P concentration in mycorrhizal plants plotted against (c) extent of root colonisation by arbuscular mycorrhizal fungi and (d) frequency of arbuscules. In panes a–b, data points represent means of plant trait data from 3–5 replicates of selected lines from Avalon × Cadenza DH wheat (*Triticum aestivum* L.) mapping population. In panes c–d, data points represent individual replicates from selected lines (see Table S5 for details of lines used)

There was no correlation between the extent of root length colonisation and the shoot MGR (Figure 6a). Similarly, there was no correlation between the frequency of arbuscules and the shoot MGR (Figure 6b). There was also no correlation between root length colonised and the shoot P concentration in mycorrhizal plants (Figure 6c) or between arbuscule frequency and shoot P concentration (Figure 6d). Vesicle frequency did not correlate with shoot MGR (Figure S8a) or shoot P concentration in mycorrhizal plants (Figure S8b). Shoot P content in mycorrhizal plants was not correlated with root length colonisation (Figure S8c), arbuscule frequency (Figure S8d) or vesicle frequency (Figure S8e).

There was no association between root dry biomass in mycorrhizal plants and the root length colonised (Figure S9a), arbuscule frequency (Figure S9b) or vesicle frequency (Figure S9c). Similarly in the mycorrhizal treatment, there was no association between shoot dry biomass and the root length colonised (Figure S9d), the arbuscule frequency (Figure S9e) or vesicle frequency (Figure S9f). Neither the extent of root length colonisation nor the arbuscule frequency in the mycorrhizal treatment was correlated with shoot P concentration (Figure S10a, c) or content (Figure S10b, d) in the non‐mycorrhizal plants. P uptake by non‐mycorrhizal plants was a good predictor of the effect of inoculation on P uptake; there were significant negative correlations between the shoot P concentration in the non‐mycorrhizal plants and the MPR (concentration (Figure S10e)), as well as between the shoot P content in the non‐mycorrhizal plants and the shoot MPR (content (Figure S10f)). However, P uptake by non‐mycorrhizal plants was a poor predictor of how beneficial AM inoculation would be for plant growth; there were no correlations between the shoot P concentration (Figure S10g) or content (Figure S10h) in the non‐mycorrhizal plants and the shoot MGR. This probably represents an unavoidable trade‐off between growth and nutrient accumulation—plants with lower biomass in the non‐mycorrhizal state are likely to have higher shoot P concentration than larger plants, and these lines will be unable to further increase their P acquisition to increase P concentration (Figures 5, S6 and S11a,b).

The QTL analysis identified six QTL statistically significantly associated with four aspects of mycorrhizal growth response. Shoot height response to inoculation was represented by only one QTL, while root dry biomass and root weight ratio were each associated with three QTL (Table 1). Logarithm of the Odds (LOD) scores varied between 3.1 and 4.4 for the identified QTL, the largest of which was associated with mycorrhizal responsiveness in total dry biomass on chromosome 6B. Four QTL were found on the B genome, two were found on the D genome and none was found on the A genome. Avalon and Cadenza were each the donor of 3 increasing alleles of the identified QTL (Table 1).

**TABLE 1:** Mycorrhizal growth response quantitative trait loci (QTL) mapping results, showing chromosome identity, trait affected, logarithm of the odds (LOD) score, identity of allele carrying the high value for relevant trait and the degree of variation explained by these QTL

QTL	Chromosome	Trait	LOD	High value allele	Variation explained
1	1B	Root dry biomass	3.5	Cadenza	13%
2	2B	Root dry biomass	4.2	Cadenza	15%
		Root weight ratio	3.2	Cadenza	11%
3	4D	Root weight ratio	4.0	Avalon	14%
4	6B	Root dry biomass	3.1	Avalon	11%
		Total dry biomass	4.4	Avalon	15%
5	7B	Root weight ratio	3.1	Cadenza	10%
6	7D	Shoot height	3.1	Avalon	11%

## 4 DISCUSSION

Our data indicate that modern, elite wheat cultivars contain sufficient genetic diversity to allow selective breeding to improve mycorrhizal growth responses. QTL associated with mycorrhizal responsiveness have been identified in several crop species, including onions (Galvan et al., 2011) and maize (Kaeppler et al., 2000). We have identified a number of QTL associated with mycorrhizal growth response in several plant traits (Table 1). Optimising the symbiosis through plant breeding will be an important contribution to ‘agro‐engineering’—an approach aimed at improving agricultural sustainability (Rowe et al., 2016; Withers et al., 2017), in part by minimising fertiliser inputs and maximising nutrient acquisition efficiency in crops.

Improving crop growth responses to AM fungi through conventional cereal breeding will rely upon varied growth and nutritional responses to inoculation among genotypes of target crops. Such variation has been observed many times in a range of species, including maize (Chu et al., 2013; Kaeppler et al., 2000; Sawers et al., 2017), rice (Diedhiou et al., 2016), barley (Baon et al., 1993; Mutairi et al., 2020), sorghum (Watts‐Williams et al., 2019) and wheat (Hetrick et al., 1992, 1993; Lehnert et al., 2018). Generally, these studies use too few genotypes to allow identification of genetic markers associated with mycorrhizal growth responsiveness by QTL analysis or genome‐wide association studies. Notably, Lehnert et al. (2018) used 94 genotypes of wheat; the diverse population of genotypes used represent 21 different countries of origin and range in age from 5 to at least 70 years since development of the variety.

By contrast, we employed a panel of doubled‐haploid lines developed from a cross of a single pair of parent cultivars, Avalon and Cadenza. As a result, the population used here contains significantly less genetic variation than in the material used by Lehnert et al. (2018). Despite using a closely related population, we still observe dramatically segregating phenotypes among lines in numerous plant growth traits. The Avalon × Cadenza doubled‐haploid mapping population studied here has previously been used for QTL studies of several traits such as grain morphology (Gegas et al., 2010), plant height (Griffiths et al., 2012), seedling rooting (Bai et al., 2013), traits associated with lodging (Piñera‐Chavez et al., 2021) and other wider agronomic traits (Amalova et al., 2021). Avalon and Cadenza have previously also been shown to experience contrasting nutrient uptake following mycorrhizal inoculation (Elliott et al., 2021). An extensive marker map for the Avalon x Cadenza population exists in the public domain, making this a useful tool for quantitative genetic analysis of wheat traits (Wang et al., 2014; http://www.wgin.org.uk/).

In a genome‐wide association study, Lehnert et al. (2018) identified two QTL associated with mycorrhizal responsiveness, on chromosomes 3D and 7D; these QTL were linked to increased grain yield and grain number, respectively. Hetrick et al. (1991) identified chromosomes 1A, 5B, 6B, 7B, 5D and 7D from the donor cultivar Cheyenne as having a positive effect on mycorrhizal responsiveness. In common with these studies, we found one QTL on chromosome 7D also associated with positive mycorrhizal responsiveness, in this case observed in shoot height. The studies of Hetrick et al. (1991) and Lehnert et al. (2018) each found unique QTL not identified either in each other study or here. The context dependence of QTL identities is illustrated by the two QTL found by Lehnert et al. (2018) shown under droughted, but not well‐watered conditions. As far as we are aware, we also identify the first QTL for root trait responsiveness to mycorrhizal inoculation (Table 1). Further data on mycorrhizal responsiveness in the Avalon x Cadenza DH mapping population from contrasting environments are now required to validate the QTL identified here, allowing the identification of candidate genes associated with mycorrhizal responsiveness.

We show that overall, inoculation with AM fungi substantially increased wheat growth across the population of lines used here, supporting the findings of previous meta‐analyses (Lehmann et al., 2012; Pellegrino et al., 2015; Zhang et al., 2019). As our plants were harvested after only 5 weeks’ growth, it is unclear whether these lines would show greater or lesser variability in mycorrhizal growth response if taken to yield, and this remains a clear priority for future phenotyping work in crop mycorrhizas. The ability of mycorrhizal plants to maintain shoot P concentrations at increased biomass, where non‐mycorrhizal counterparts showed a relative dilution (Figures 5 and S6), might be expected, given the well‐established role of AM fungi in enhancing plant P acquisition (Bolan, 1991; Bolan et al., 1983; Smith et al., 2011). Strong negative correlations between shoot P concentration in the non‐mycorrhizal state and subsequent shoot P concentration response to inoculation (Figure S10e–f) show that as with biomass, AM fungal inoculation most strongly benefits those lines which perform relatively poorly in the non‐mycorrhizal state. Although testing the response of these lines under low‐P conditions was beyond the scope of this study, it is interesting to note that the shoot P concentration in the non‐mycorrhizal state was not correlated with the extent to which these lines become colonised by AM fungi (Figure S10a–d). If mycorrhizal responsiveness was purely controlled by the extent of mycorrhizal colonisation, those lines with low P in the non‐mycorrhizal state might be expected to have greater colonisation in the mycorrhizal treatment, but this was not seen. These data suggest mycorrhizal benefit in these lines was not correlated with the extent of mycorrhizal colonisation (Figures 6a–d, S8a–e and S9a–f). A lack of correlation between P content or concentration in the non‐mycorrhizal state and the MGR (Figure S10g–h) is perhaps not surprising, given that, as demonstrated in Figure 5, shoot P concentration in the non‐mycorrhizal state correlates negatively with shoot biomass. Smaller plants showed higher shoot P concentration, while they are also likely to positive biomass response to inoculation (Figure 3). A significant correlation between MGR (shoot biomass) and MPR (content) suggests lines which receive a benefit from inoculation in terms of P uptake are also likely to see this translated into increased biomass (Figure S11b). A lack of correlation between MPR (concentration) and MGR (shoot biomass (Figure S11a)) suggests a trade‐off; plants are unable to substantially increase biomass and P concentration concurrently.

A number of QTL associated with the extent of mycorrhizal fungal colonisation have recently been identified in crops including tomato (Plouznikoff et al., 2019), soya bean (Pawlowski et al., 2020), rice (Davidson et al., 2019), durum wheat (De Vita et al., 2018) and bread wheat (Lehnert et al., 2017). Although a certain degree of colonisation is presumably required for substantial mycorrhizal benefit, the degree to which plants respond positively to mycorrhizal inoculation or colonisation is often not correlated with intraradical fungal biomass or the frequency of arbuscules (Martin et al., 2012; Sawers et al., 2017; Smith et al., 2004; Thirkell et al., 2019; Thirkell et al., 2021; Pawlowski et al., 2020; but see Huang et al., 2020). In a meta‐analysis, Treseder (2013) did find that colonisation levels were correlated with both mycorrhizal growth response and P response, but the association was notably weak. We found no correlation between mycorrhizal colonisation and AM fungal benefit to plant growth or P uptake (Figures S7 and S8b–e). Furthermore, if the root length colonised and the P benefit were tightly coupled, we might expect to have seen negative correlations between P content or concentration of shoots in non‐mycorrhizal plants and levels of AM fungal colonisation in the corresponding mycorrhizal group of the same lines, but these trends were not apparent (Figure S10a–d). We suggest factors other than colonisation levels are likely to exhibit significant control over plant response to colonisation (Lefebvre, 2020; Ramírez‐Flores et al., 2020); focussing solely on maximising AM fungal colonisation is a potentially risky strategy to improve crop responsiveness. High levels of mycorrhizal colonisation may even elicit negative growth responses (Ryan et al., 2005; Tran et al., 2019). Levels of root length colonisation here appear relatively high after only 5 weeks’ growth, although using a simple growth medium will have reduced the capacity for competing microbes to colonise the wheat roots. The levels of colonisation here are comparable with those found using similar wheat genotypes and inocula in previous experiments (Elliott et al., 2021; Thirkell et al., 2020).

Maximising the proliferation of root‐external hyphae may have a greater influence over mycorrhizal responsiveness than maximising root length colonisation (Diedhiou et al., 2016; Munkvold et al., 2004; Sawers et al., 2017). This is an appealing prospect, as the extraradical hyphae are largely responsible for the principal benefit of the symbiosis, that of increased P acquisition from the soil (Smith & Read, 2008). Increasing quantities of external hyphae may represent another target for breeders, as crop genotype has been shown to influence this trait (Sawers et al., 2017). Fungal genotype also significantly affects root‐external hyphae—shifting the AM fungal community to encourage colonisation by fungal species which have an edaphophilic rather than rhizophilic growth habit (Han et al., 2020) may also be beneficial. Mycorrhizal fungal species and isolates have highly variable effects on plant nutrition and growth (Klironomos, 2003; Mensah et al., 2015; Munkvold et al., 2004; Watts‐Williams et al., 2019). Beneficial shifts in AM fungal community composition in arable soils may be achievable through cereal breeding or agronomic practices, but this remains to be investigated (Thirkell et al., 2021). Low AM fungal diversity in arable soils may limit the potential for shifts in intraradical AM fungal communities towards more beneficial assemblages (Schneider et al., 2015; Schnoor et al., 2011). Fully exploiting mycorrhizal symbioses in arable crops will likely require a combination of agronomic and breeding innovation.

It is important to note that our inoculum comprised a mix of 5 species of AM fungi—without molecular identification we cannot comment on the diversity or evenness of the intraradical community here. The extent to which cereal crop genotype influences intraradical AM fungal community composition is unclear (Aguilera et al., 2014; Parvin et al., 2021; Stefani et al., 2020). Conceivably, lines showing negative response to AM fungal inoculation could have intraradical fungal communities dominated by different fungal genotypes than those found in positive responding lines. Fungal identity seems to be unimportant in some cases—fungal colonisation of any kind may be more influential (Walder & van der Heijden, 2015). An evolutionarily conserved plant signalling pathway which predisposes positive responsiveness following AM fungal colonisation would be of obvious utility; as yet, no such pathway has been identified. Shared signalling pathways appear to regulate colonisation by symbionts and some pathogenic microbes (Güimil et al., 2005; Wang, Basten, & Zeng, 2012; Wang, Schornack, et al., 2012; Zipfel & Oldroyd, 2017), and plants must distinguish between these organisms. Trade‐offs between susceptibility to colonisation by pathogens and symbionts (e.g. AM fungi) are perhaps therefore necessary and may limit the extent of mycorrhizal colonisation (Jacott et al., 2017).

Key to understanding whether it is possible to harness the AM symbiosis in industrial agricultural systems will be to determine which factors allow some elite lines to respond positively to AM colonisation, rather than exclusively those which show poor performance in the non‐mycorrhizal state (Janos, 2007). Studies in quantitative genetics and crop physiology are now required to achieve this. Here, panels of closely related crop genotypes showing divergent responses to AM fungal inoculation, such as the Avalon × Cadenza mapping population, will likely prove invaluable.

Root epidermal phosphate transporters remain an important target for improving P uptake efficiencies in cereals (Wang et al., 2016) and may be central to understanding varied mycorrhizal responsiveness. A clear target for improving plant responsiveness to mycorrhizal colonisation will be to attempt to prevent plant P transporter downregulation by mycorrhizal colonisation, so that the mycorrhizal and direct P uptake pathways are additive rather than substitutive (Smith et al., 2011). Whether plant root N transporters are similarly affected by mycorrhizal colonisation is not clear (Duan et al., 2015; Tian et al., 2017). The influence of root system architecture and root hair morphology on mycorrhizal responsiveness is less clear among closely related genotypes of crops than among unrelated wild species (Hetrick, 1991; Hetrick et al., 1991; Maherali, 2014; Yang et al., 2015). AM fungi can offer substantial benefit to barley mutants in which root hairs are small in size or number (Jakobsen et al., 2005). Determining the effect of root characteristics on mycorrhizal responsiveness will be an important step in identifying target traits to allow exploitation of mycorrhizas through crop breeding.

By distinguishing between mycorrhizal responsiveness and dependence, it has been argued (Janos, 2007; Sawers et al., 2008) that modern cereals perform far better than their ancestors or wild progenitors in the non‐mycorrhizal state, so the capacity for mycorrhizal responsiveness in yield or nutrition is reduced. Characterising the mechanistic nature of how mycorrhizas may influence crop traits should ensure that any developments in breeding or agronomy to improve mycorrhizal benefit will not simply substitute for gains which have been made in improving innate plant traits, such as rooting architecture, nutrient allocation or plant defence responses. Developments which substitute mycorrhizal mechanisms for plant mechanisms may however be beneficial where they reduce the carbon cost while maintaining or improving crop mineral nutrient uptake, or reduce the demand for fertiliser application. Further potential benefits to cereal hosts from the symbiosis must also be examined when considering whether these substitutions are worthwhile (Rillig et al., 2019; Ryan et al., 2019). Positive effects at the greater spatial or temporal scales, such as carbon sequestration, nutrient retention and soil stability, also require investigation. Selecting cereal genotypes for more positive mycorrhizal responsiveness may also select for enhancements in these wider ecosystem benefits, although this remains to be tested. If yields can be maintained or even improved through the fostering of more beneficial mycorrhizal associations while also improving sustainability, trade‐offs between yield and sustainability may be avoided.

## CONFLICT OF INTEREST

The authors declare no conflict of interest.

## AUTHOR CONTRIBUTIONS

TJT, NP, LJ, TD and KJF designed the study; TJT, DP, TA and AE conducted the experimental work; TJT, LJ, MG, NP, KJF and TD interpreted results, TJT conducted data analysis and wrote the first draft manuscript. All authors approved the submitted version of the manuscript.

## Supporting information

Appendix S1

## References

[fes3370-bib-0001] Aguilera, P. , Cornejo, P. , Borie, F. , Barea, J. M. , von Baer, E. , & Oehl, F. (2014). Diversity of arbuscular mycorrhizal fungi associated with *Triticum aestivum* L. plants growing in an Andosol with high aluminum level. Agriculture, Ecosystems & Environment, 186, 178–184. 10.1016/j.agee.2014.01.029

[fes3370-bib-0002] Amalova, A. , Abugalieva, S. , Chudinov, V. , Sereda, G. , Tokhetova, L. , Abdikhalyk, A. , & Turuspekov, Y. (2021). QTL mapping of agronomic traits in wheat using the UK Avalon × Cadenza reference mapping population grown in Kazakhstan. PeerJ, 9, e10733. 10.7717/peerj.10733 33643705 PMC7897413

[fes3370-bib-0003] Bai, C. , Liang, Y. , & Hawkesford, M. J. (2013). Identification of QTLs associated with seedling root traits and their correlation with plant height in wheat. Journal of Experimental Botany, 64, 1745–1753. 10.1093/jxb/ert041 23564959 PMC3617839

[fes3370-bib-0004] Baon, J. B. , Smith, S. E. , & Alston, A. M. (1993). Mycorrhizal responses of barley cultivars differing in P efficiency. Plant and Soil, 157, 97–105. 10.1007/BF00038752

[fes3370-bib-0005] Berger, F. , & Gutjahr, C. (2021). Factors affecting plant responsiveness to arbuscular mycorrhiza. Current Opinion in Plant Biology, 59, 101994. 10.1016/j.pbi.2020.101994 33450718

[fes3370-bib-0006] Bolan, N. S. (1991). A critical review on the role of mycorrhizal fungi in the uptake of phosphorus by plants. Plant and Soil, 134, 189–207. 10.1007/BF00012037

[fes3370-bib-0007] Bolan, N. S. , Robson, A. D. , & Barrow, N. J. (1983). Plant and soil factors including mycorrhizal infection causing sigmoidal response of plants to applied phosphorus. Plant and Soil, 73, 187–201. 10.1007/BF02197715

[fes3370-bib-0008] Cameron, D. D. , Neal, A. L. , van Wees, S. C. M. , & Ton, J. (2013). Mycorrhiza‐induced resistance: more than the sum of its parts? Trends in Plant Science, 18, 539–545. 10.1016/j.tplants.2013.06.004 23871659 PMC4194313

[fes3370-bib-0009] Chu, Q. , Wang, X. , Yang, Y. , Chen, F. , Zhang, F. , & Feng, G. (2013). Mycorrhizal responsiveness of maize (*Zea mays* L.) genotypes as related to releasing date and available P content in soil. Mycorrhiza, 23(6), 497–505. 10.1007/s00572-013-0492-0 23503868

[fes3370-bib-0010] Cordell, D. , & White, S. (2014). Life's bottleneck: sustaining the world's phosphorus for a food secure future. Annual Review of Environment and Resources, 39, 161–188. 10.1146/annurev-environ-010212-113300

[fes3370-bib-0011] Davidson, H. , Shrestha, R. , Cornulier, T. , Douglas, A. , Travis, T. , Johnson, D. , & Price, A. H. (2019). Spatial effects and GWA mapping of root colonization assessed in the interaction between the rice diversity panel 1 and an arbuscular mycorrhizal fungus. Frontiers in Plant Science, 10(633), 1–14. 10.3389/fpls.2019.00633 31156686 PMC6533530

[fes3370-bib-0012] De Vita, P. , Avio, L. , Sbrana, C. , Laido, G. , Marone, D. , Mastrangelo, A. M. , Cattivelli, L. , & Giovannetti, M. (2018). Genetic markers associated to arbuscular mycorrhizal colonization in durum wheat. Scientific Reports, 8, 1–12. 10.1038/s41598-018-29020-6 30006562 PMC6045686

[fes3370-bib-0013] Diedhiou, A. G. , Mbaye, F. K. , Mbodj, D. , Faye, M. N. , Pignoly, S. , Ndoye, I. , Djaman, K. , Gaye, S. , Kane, A. , Laplaze, L. , Manneh, B. , & Champion, A. (2016). Field trials reveal ecotype‐specific responses to mycorrhizal inoculation in rice. PLoS One, 11, e0167014. 10.1371/journal.pone.0167014 27907023 PMC5132163

[fes3370-bib-0014] Duan, J. , Tian, H. , Drijber, R. A. , & Gao, Y. (2015). Systemic and local regulation of phosphate and nitrogen transporter genes by arbuscular mycorrhizal fungi in roots of winter wheat (*Triticum aestivum* L.). Plant Physiology and Biochemistry, 96, 199–208. 10.1016/j.plaphy.2015.08.006 26298806

[fes3370-bib-0015] Elliott, A. J. , Daniell, T. J. , Cameron, D. D. , & Field, K. J. (2021). A commercial arbuscular mycorrhizal inoculum increases root colonization across wheat cultivars but does not increase assimilation of mycorrhiza‐acquired nutrients. Plants, People, Planet, 3, 588–599. 10.1002/ppp3.10094 34853824 PMC8607474

[fes3370-bib-0016] Elser, J. , & Bennett, E. (2011). A broken biogeochemical cycle. Nature, 478, 29–31. 10.1038/478029a 21979027

[fes3370-bib-0017] Galvan, G. A. , Kuyper, T. W. , Burger, K. , Keizer, L. C. P. , Hoekstra, R. F. , Kik, C. , & Scholten, O. E. (2011). Genetic analysis of the interaction between *Allium* species and arbuscular mycorrhizal fungi. Theoretical and Applied Genetics, 122, 947–960. 10.1007/s00122-010-1501-8 21222096 PMC3043257

[fes3370-bib-0018] Gegas, V. C. , Nazari, A. , Griffiths, S. , Simmonds, J. , Fish, L. , Orford, S. , Sayers, L. , Doonan, J. H. , & Snape, J. W. (2010). A genetic framework for grain size and shape variation in wheat. The Plant Cell, 22, 1046–1056. 10.1105/tpc.110.074153 20363770 PMC2879751

[fes3370-bib-0019] Grassini, P. , Eskridge, K. M. , & Cassman, K. G. (2013). Distinguishing between yield advances and yield plateaus in historical crop production trends. Nature Communications, 4, 1–11. 10.1038/ncomms3918 PMC3905725 24346131

[fes3370-bib-0020] Griffiths, S. , Simmonds, J. , Leverington, M. , Wang, Y. , Fish, L. , Sayers, L. , Alibert, L. , Orford, S. , Wingen, L. , & Snape, J. (2012). Meta‐QTL analysis of the genetic control of crop height in elite European winter wheat germplasm. Molecular Breeding, 29, 159–171. 10.1007/s11032-010-9534-x 19430758

[fes3370-bib-0021] Güimil, S. , Chang, H.‐S. , Zhu, T. , Sesma, A. , Osbourn, A. , Roux, C. , Ioannidis, V. , Oakeley, E. J. , Docquier, M. , Descombes, P. , Briggs, S. , & Paskowski, U. (2005). Comparative transcriptomics of rice reveals an ancient pattern of response to microbial colonization. Proceedings of the National Academy of Sciences of the United States of America, 102, 8066–8070. 10.1073/pnas.0502999102 15905328 PMC1142390

[fes3370-bib-0022] Han, Y. , Feng, J. , Han, M. , & Zhu, B. (2020). Responses of arbuscular mycorrhizal fungi to nitrogen addition: a meta‐analysis. Global Change Biology, 26, 7229–7241. 10.1111/gcb.15369 32981218

[fes3370-bib-0023] Hetrick, B. A. D. (1991). Mycorrhizas and root architecture. Experientia, 47, 355–362. 10.1007/BF01972077

[fes3370-bib-0024] Hetrick, B. A. D. , Wilson, G. W. T. , & Cox, T. S. (1992). Mycorrhizal dependence of modern wheat varieties, landraces and ancestors. Canadian Journal of Botany, 70, 2032–2040. 10.1139/b92-253

[fes3370-bib-0025] Hetrick, B. A. D. , Wilson, G. W. T. , & Cox, T. S. (1993). Mycorrhizal dependence of modern wheat cultivars and ancestors: a synthesis. Canadian Journal of Botany, 71, 512–518. 10.1139/b93-056

[fes3370-bib-0026] Hetrick, B. A. D. , Wilson, G. W. T. , & Leslie, J. F. (1991). Root architecture of warm‐ and cool‐season grasses: relationship to mycorrhizal dependence. Canadian Journal of Botany, 1, 112–118. 10.1139/b91-016

[fes3370-bib-0027] Huang, R. , Li, Z. , Mao, C. , Zhang, H. , Sun, Z. , Li, H. , Huang, C. , Feng, Y. , Shen, X. , & Bucher, M. (2020). Natural variation at OsCERK1 regulates arbuscular mycorrhizal symbiosis in rice. New Phytologist, 225, 1762–1776. 10.1111/nph.16158 31484206

[fes3370-bib-0028] Jacott, C. N. , Murray, J. D. , & Ridout, C. J. (2017). Trade‐offs in arbuscular mycorrhizal symbiosis: disease resistance, growth responses and perspectives for crop breeding. Agronomy, 7, 75. 10.3390/agronomy7040075

[fes3370-bib-0029] Jakobsen, I. , Chen, B. , Munkvold, L. , Lundsgaard, T. , & Zhu, Y.‐G. (2005). Contrasting phosphate acquisition of mycorrhizal fungi with that of root hairs using the root hairless barley mutant. Plant, Cell & Environment, 28, 928–938. 10.1111/j.1365-3040.2005.01345.x

[fes3370-bib-0030] Janos, D. P. (2007). Plant responsiveness to mycorrhizas differs from dependence upon mycorrhizas. Mycorrhiza, 17, 75–91. 10.1007/s00572-006-0094-1 17216499

[fes3370-bib-0031] Kaeppler, S. M. , Parke, J. L. , Mueller, S. M. , Senior, L. , Stuber, C. , & Tracy, W. F. (2000). Variation among maize inbred lines and detection of quantitative trait loci for growth at low phosphorus and responsiveness to arbuscular mycorrhizal fungi. Crop Science, 40, 358–364. 10.2135/cropsci2000.402358x

[fes3370-bib-0032] Klironomos, J. N. (2003). Variation in plant response to native and exotic arbuscular mycorrhizal fungi. Ecology, 84, 2292–2301. 10.1890/02-0413

[fes3370-bib-0033] Kvakić, M. , Pellerin, S. , Ciais, P. , Achat, D. L. , Augusto, L. , Denoroy, P. , Gerber, J. S. , Goll, D. , Mollier, A. , Mueller, N. D. , Wang, X. , & Ringeval, B. (2018). Quantifying the limitation to world cereal production due to soil phosphorus status. Global Biogeochemical Cycles, 32, 143–157. 10.1002/2017GB005754

[fes3370-bib-0034] LaCanne, C. E. , & Lundgren, J. G. (2018). Regenerative agriculture: merging farming and natural resource conservation profitably. PeerJ, 6, e4428. 10.7717/peerj.4428 29503771 PMC5831153

[fes3370-bib-0035] Lefebvre, B. (2020). An opportunity to breed rice for improved benefits from the arbuscular mycorrhizal symbiosis? New Phytologist, 225, 1404–1406. 10.1111/nph.16333 31823373

[fes3370-bib-0036] Lehmann, A. , Barto, E. K. , Powell, J. R. , & Rillig, M. C. (2012). Mycorrhizal responsiveness trends in annual crop plants and their wild relatives‐a meta‐analysis on studies from 1981 to 2010. Plant and Soil, 355, 231–250. 10.1007/s11104-011-1095-1

[fes3370-bib-0037] Lehnert, H. , Serfling, A. , Enders, M. , Friedt, W. , & Ordon, F. (2017). Genetics of mycorrhizal symbiosis in winter wheat (*Triticum aestivum*). New Phytologist, 215, 779–791. 10.1111/nph.14595 28517039

[fes3370-bib-0038] Lehnert, H. , Serfling, A. , Friedt, W. , & Ordon, F. (2018). Genome‐wide association studies reveal genomic regions associated with the response of wheat (*Triticum aestivum* L.) to mycorrhizae under drought stress conditions. Frontiers in Plant Science, 9, 1–24. 10.3389/fpls.2018.01728 30568663 PMC6290350

[fes3370-bib-0039] Luginbuehl, L. H. , Menard, G. N. , Kurup, S. , Van Erp, H. , Radhakrishnan, G. V. , Breakspear, A. , Oldroyd, G. E. D. , & Eastmond, P. J. (2017). Fatty acids in arbuscular mycorrhizal fungi are synthesized by the host plant. Science, 356, 1175–1178. 10.1126/science.aan0081 28596311

[fes3370-bib-0040] Maherali, H. (2014). Is there an association between root architecture and mycorrhizal growth response? New Phytologist, 204, 192–200. 10.1111/nph.12927 25041241

[fes3370-bib-0041] Martin, S. L. , Mooney, S. J. , Dickinson, M. J. , & West, H. M. (2012). The effects of simultaneous root colonisation by three *Glomus* species on soil pore characteristics. Soil Biology and Biochemistry, 49, 167–173. 10.1016/j.soilbio.2012.02.036

[fes3370-bib-0042] McGonigle, T. P. , Miller, M. H. , Evans, D. G. , Fairchild, G. L. , & Swan, J. A. (1990). A new method which gives an objective measure of colonization of roots by vesicular arbuscular mycorrhizal fungi. New Phytologist, 115, 495–501. 10.1111/j.1469-8137.1990.tb00476.x 33874272

[fes3370-bib-0043] McMillan, V. E. , Gutteridge, R. J. , & Hammond‐Kosack, K. E. (2014). Identifying variation in resistance to the take‐all fungus, *Gaeumannomyces graminis* var. tritici, between different ancestral and modern wheat species. BMC Plant Biology, 14, 212. 10.1186/s12870-014-0212-8 25084989 PMC4236718

[fes3370-bib-0044] Mensah, J. A. , Koch, A. M. , Antunes, P. M. , Kiers, E. T. , Hart, M. , & Bücking, H. (2015). High functional diversity within species of arbuscular mycorrhizal fungi is associated with differences in phosphate and nitrogen uptake and fungal phosphate metabolism. Mycorrhiza, 25, 533–546. 10.1007/s00572-015-0631-x 25708401

[fes3370-bib-0045] Morrell, P. L. , Buckler, E. S. , & Ross‐Ibarra, J. (2012). Crop genomics: Advances and applications. Nature Reviews Genetics, 13, 85–96. 10.1038/nrg3097 22207165

[fes3370-bib-0046] Munkvold, L. , Kjøller, R. , Vestberg, M. , Rosendahl, S. , & Jakobsen, I. (2004). High functional diversity within species of arbuscular mycorrhizal fungi. New Phytologist, 164, 357–364. 10.1111/j.1469-8137.2004.01169.x 33873553

[fes3370-bib-0047] Murphy, J. , & Riley, J. P. (1962). A modified single solution method for the determination of phosphate in natural waters. Analytica Chimica Acta, 27, 31–36. 10.1016/S0003-2670(00)88444-5

[fes3370-bib-0048] Mutairi, A. A. A. , Cavagnaro, T. R. , Khor, S. F. , Neumann, K. , Burton, R. A. , & Watts‐Williams, S. J. (2020). The effect of zinc fertilisation and arbuscular mycorrhizal fungi on grain quality and yield of contrasting barley cultivars. Functional Plant Biology, 47, 122–133. 10.1071/FP19220 31910148

[fes3370-bib-0049] Parvin, S. , Van Geel, M. , Ali, M. M. , Yeasmin, T. , Lievens, B. , & Honnay, O. (2021). A comparison of the arbuscular mycorrhizal fungal communities among Bangladeshi modern high yielding and traditional rice varieties. Plant and Soil, 462(1–2), 109–124. 10.1007/s11104-021-04858-4

[fes3370-bib-0050] Pawlowski, M. L. , Vuong, T. D. , Valliyodan, B. , Nguyen, H. T. , & Hartman, G. L. (2020). Whole‐genome resequencing identifies quantitative trait loci associated with mycorrhizal colonization of soybean. Theoretical and Applied Genetics, 133, 409–417. 10.1007/s00122-019-03471-5 31707439

[fes3370-bib-0051] Pellegrino, E. , Öpik, M. , Bonari, E. , & Ercoli, L. (2015). Responses of wheat to arbuscular mycorrhizal fungi: a meta‐analysis of field studies from 1975 to 2013. Soil Biology and Biochemistry, 84, 210–217. 10.1016/j.soilbio.2015.02.020

[fes3370-bib-0052] Piñera‐Chavez, F. J. , Berry, P. M. , Foulkes, M. J. , Sukumaran, S. , & Reynolds, M. P. (2021). Identifying QTLs for lodging‐associated traits in the wheat doubled‐haploid population Avalon × Cadenza. Crop Science, 61(4), 2371–2386. 10.1002/csc2.20485

[fes3370-bib-0053] Pingali, P. L. (2012). Green revolution: Impacts, limits, and the path ahead. Proceedings of the National Academy of Sciences, 109, 12302–12308. 10.1073/pnas.0912953109 PMC3411969 22826253

[fes3370-bib-0054] Plouznikoff, K. , Asins, M. J. , de Boulois, H. D. , Carbonell, E. A. , & Declerck, S. (2019). Genetic analysis of tomato root colonization by arbuscular mycorrhizal fungi. Annals of Botany, 124, 933–946. 10.1093/aob/mcy240 30753410 PMC7145532

[fes3370-bib-0055] R Core Team (2020). R: A language and environment for statistical computing. R Foundation for Statistical Computing.

[fes3370-bib-0056] Ramírez‐Flores, M. R. , Perez‐Limon, S. , Li, M. , Barrales‐Gamez, B. , Albinsky, D. , Paszkowski, U. , Olalde‐Portugal, V. , & Sawers, R. J. H. (2020). The genetic architecture of host response reveals the importance of arbuscular mycorrhizae to maize cultivation. eLife, 9, e61701. 10.7554/eLife.61701 33211006 PMC7676867

[fes3370-bib-0057] Raven, J. A. , Lambers, H. , Smith, S. E. , & Westoby, M. (2018). Costs of acquiring phosphorus by vascular land plants: patterns and implications for plant coexistence. New Phytologist, 217, 1420–1427. 10.1111/nph.14967 29292829

[fes3370-bib-0058] Reganold, J. P. , & Wachter, J. M. (2016). Organic agriculture in the twenty‐first century. Nature Plants, 2, 15221. 10.1038/nplants.2015.221 27249193

[fes3370-bib-0059] Rillig, M. C. , Aguilar‐Trigueros, C. A. , Camenzind, T. , Cavagnaro, T. R. , Degrune, F. , Hohmann, P. , Lammel, D. R. , Mansour, I. , Roy, J. , Heijden, M. G. A. , & Yang, G. (2019). Why farmers should manage the arbuscular mycorrhizal symbiosis. New Phytologist, 222, 1171–1175. 10.1111/nph.15602 30657593

[fes3370-bib-0060] Rillig, M. C. , Sosa‐Hernandez, M. A. , Roy, J. , Aguilar‐Trigueros, C. A. , Valyi, K. , & Lehmann, A. (2016). Towards an integrated mycorrhizal technology: harnessing mycorrhiza for sustainable intensification in agriculture. Frontiers in Plant Science, 7(1625), 1–5. 10.3389/fpls.2016.01625 27833637 PMC5081377

[fes3370-bib-0061] Rowe, H. , Withers, P. J. A. , Baas, P. , Chan, N. I. , Doody, D. , Holiman, J. , Jacobs, B. , Li, H. , MacDonald, G. K. , McDowell, R. , Sharpley, A. , Shen, K. , Taheri, W. , Wallenstein, M. , & Weintraub, M. (2016). Integrating legacy soil phosphorus into sustainable nutrient management strategies for future food, bioenergy and water security. Nutrient Cycling in Agroecosystems, 104, 393–412. 10.1007/s10705-015-9726-1

[fes3370-bib-0062] RStudio Team (2015). RStudio: Integrated development for R. RStudio.

[fes3370-bib-0063] Ryan, M. H. , & Graham, J. H. (2018). Little evidence that farmers should consider abundance or diversity of arbuscular mycorrhizal fungi when managing crops. New Phytologist, 220, 1092–1107. 10.1111/nph.15308 29987890

[fes3370-bib-0064] Ryan, M. H. , Graham, J. H. , Morton, J. B. , & Kirkegaard, J. A. (2019). Research must use a systems agronomy approach if management of the arbuscular mycorrhizal symbiosis is to contribute to sustainable intensification. New Phytologist, 222, 1176–1178. 10.1111/nph.15600 30657177

[fes3370-bib-0065] Ryan, M. H. , Van Herwaarden, A. F. , Angus, J. F. , & Kirkegaard, J. A. (2005). Reduced growth of autumn‐sown wheat in a low‐P soil is associated with high colonisation by arbuscular mycorrhizal fungi. Plant and Soil, 270, 275–286. 10.1007/s11104-004-1611-7

[fes3370-bib-0066] Sawers, R. J. H. , Gutjahr, C. , & Paszkowski, U. (2008). Cereal mycorrhiza: an ancient symbiosis in modern agriculture. Trends in Plant Science, 13, 93–97. 10.1016/j.tplants.2007.11.006 18262822

[fes3370-bib-0067] Sawers, R. J. H. , Svane, S. F. , Quan, C. , Grønlund, M. , Wozniak, B. , Gebreselassie, M.‐N. , González‐Muñoz, E. , Chávez Montes, R. A. , Baxter, I. , Goudet, J. , Jakobsen, I. , & Paszkowski, U. (2017). Phosphorus acquisition efficiency in arbuscular mycorrhizal maize is correlated with the abundance of root‐external hyphae and the accumulation of transcripts encoding PHT1 phosphate transporters. New Phytologist, 214, 632–643. 10.1111/nph.14403 28098948

[fes3370-bib-0068] Schneider, K. D. , Lynch, D. H. , Dunfield, K. , Khosla, K. , Jansa, J. , & Voroney, R. P. (2015). Farm system management affects community structure of arbuscular mycorrhizal fungi. Applied Soil Ecology, 96, 192–200. 10.1016/j.apsoil.2015.07.015

[fes3370-bib-0069] Schnoor, T. K. , Lekberg, Y. , Rosendahl, S. , & Olsson, P. A. (2011). Mechanical soil disturbance as a determinant of arbuscular mycorrhizal fungal communities in semi‐natural grassland. Mycorrhiza, 21, 211–220. 10.1007/s00572-010-0325-3 20593293

[fes3370-bib-0070] Singh, A. K. , Hamel, C. , DePauw, R. M. , & Knox, R. E. (2012). Genetic variability in arbuscular mycorrhizal fungi compatibility supports the selection of durum wheat genotypes for enhancing soil ecological services and cropping systems in Canada. Canadian Journal of Microbiology, 58, 293–302. 10.1139/w11-140 22356605

[fes3370-bib-0071] Smith, F. A. , & Smith, S. E. (2011). What is the significance of the arbuscular mycorrhizal colonisation of many economically important crop plants? Plant and Soil, 348, 63–79. 10.1007/s11104-011-0865-0

[fes3370-bib-0072] Smith, S. E. , Jakobsen, I. , Grønlund, M. , & Smith, F. A. (2011). Roles of arbuscular mycorrhizas in plant phosphorus nutrition: interactions between pathways of phosphorus uptake in arbuscular mycorrhizal roots have important implications for understanding and manipulating plant phosphorus acquisition. Plant Physiology, 156, 1050–1057. 10.1104/pp.111.174581 21467213 PMC3135927

[fes3370-bib-0073] Smith, S. E. , & Read, D. J. (2008). Mycorrhizal symbiosis. Academic Press.

[fes3370-bib-0074] Smith, S. E. , Smith, F. A. , & Jakobsen, I. (2004). Functional diversity in arbuscular mycorrhizal (AM) symbioses: the contribution of the mycorrhizal P uptake pathway is not correlated with mycorrhizal responses in growth or total P uptake. New Phytologist, 162, 511–524. 10.1111/j.1469-8137.2004.01039.x

[fes3370-bib-0075] Sosa‐Hernández, M. A. , Leifheit, E. F. , Ingraffia, R. , & Rillig, M. C. (2019). Subsoil arbuscular mycorrhizal fungi for sustainability and climate‐smart agriculture: a solution right under our feet? Frontiers in Microbiology, 10, 10.3389/fmicb.2019.00744 PMC6473167 31031726

[fes3370-bib-0076] Spatafora, J. W. , Chang, Y. , Benny, G. L. , Lazarus, K. , Smith, M. E. , Berbee, M. L. , Bonito, G. , Corradi, N. , Grigoriev, I. , Gryganskyi, A. , James, T. Y. , O’Donnell, K. , Roberson, R. W. , Taylor, T. N. , Uehling, J. , Vilgalys, R. , White, M. M. , & Stajich, J. E. (2016). A phylum‐level phylogenetic classification of zygomycete fungi based on genome‐scale data. Mycologia, 108, 1028–1046. 10.3852/16-042 27738200 PMC6078412

[fes3370-bib-0077] Stefani, F. , Dupont, S. , Laterrière, M. , Knox, R. , Ruan, Y. , Hamel, C. , & Hijri, M. (2020). Similar arbuscular mycorrhizal fungal communities in 31 durum wheat cultivars (*Triticum turgidum* L. var. durum) under field conditions in Eastern Canada. Frontiers in Plant Science, 11(1206), 1–15. 10.3389/fpls.2020.01206 32849748 PMC7431883

[fes3370-bib-0078] Thirkell, T. J. , Cameron, D. D. , & Hodge, A. (2019). Contrasting nitrogen fertilisation rates alter mycorrhizal contribution to barley nutrition in a field trial. Frontiers in Plant Science, 10, 1312. 10.3389/fpls.2019.01312 31736991 PMC6831614

[fes3370-bib-0079] Thirkell, T. J. , Campbell, M. , Driver, J. , Pastok, D. , Merry, B. , & Field, K. J. (2021). Cultivar‐dependent increases in mycorrhizal nutrient acquisition by barley in response to elevated CO_2_ . Plants, People, Planet, 3(5), 553–566. 10.1002/ppp3.10174

[fes3370-bib-0080] Thirkell, T. J. , Charters, M. D. , Elliott, A. J. , Sait, S. M. , & Field, K. J. (2017). Are mycorrhizal fungi our sustainable saviours? Considerations for achieving food security. Journal of Ecology, 105, 921–929. 10.1111/1365-2745.12788

[fes3370-bib-0081] Thirkell, T. J. , Pastok, D. , & Field, K. J. (2020). Carbon for nutrient exchange between arbuscular mycorrhizal fungi and wheat varies according to cultivar and changes in atmospheric carbon dioxide concentration. Global Change Biology, 26, 1725–1738. 10.1111/gcb.14851 31645088 PMC7079082

[fes3370-bib-0082] Tian, H. , Yuan, X. , Duan, J. , Li, W. , Zhai, B. , & Gao, Y. (2017). Influence of nutrient signals and carbon allocation on the expression of phosphate and nitrogen transporter genes in winter wheat (*Triticum aestivum* L.) roots colonized by arbuscular mycorrhizal fungi. PLoS One, 12, e0172154. 10.1371/journal.pone.0172154 28207830 PMC5312871

[fes3370-bib-0083] Tinker, P. B. , & Nye, P. H. (2000). Solute Movement in the Rhizosphere. Oxford University Press.

[fes3370-bib-0084] Tran, B. T. T. , Watts‐Williams, S. J. , & Cavagnaro, T. R. (2019). Impact of an arbuscular mycorrhizal fungus on the growth and nutrition of fifteen crop and pasture plant species. Functional Plant Biology, 46, 732–742. 10.1071/FP18327 31092308

[fes3370-bib-0085] Treseder, K. K. (2013). The extent of mycorrhizal colonization of roots and its influence on plant growth and phosphorus content. Plant and Soil, 371, 1–13. 10.1007/s11104-013-1681-5

[fes3370-bib-0086] Vierheilig, H. , Coughlan, A. , Wyss, U. , & Piche, Y. (1998). Ink and vinegar, a simple staining technique for arbuscular mycorrhizal fungi. Applied and Environmental Biology, 64(12), 5004–5007. 10.1128/AEM.64.12.5004-5007.1998 PMC90956 9835596

[fes3370-bib-0087] Vitousek, P. M. , Naylor, R. , Crews, T. , David, M. B. , Drinkwater, L. E. , Holland, E. , Johnes, P. J. , Katzenberger, J. , Martinelli, L. A. , Matson, P. A. , Nziguheba, G. , Ojima, D. , Palm, C. A. , Robertson, G. P. , Sanchez, P. A. , Townsend, A. R. , & Zhang, F. S. (2009). Nutrient imbalances in agricultural development. Science, 324, 1519–1520. 10.1126/science.1170261 19541981

[fes3370-bib-0088] Walder, F. , & van der Heijden, M. G. A. (2015). Regulation of resource exchange in the arbuscular mycorrhizal symbiosis. Nature Plants, 1, 7. 10.1038/nplants.2015.159 27251530

[fes3370-bib-0089] Wang, E. T. , Schornack, S. , Marsh, J. F. , Gobbato, E. , Schwessinger, B. , Eastmond, P. , Schultze, M. , Kamoun, S. , & Oldroyd, G. E. D. (2012). A common signaling process that promotes mycorrhizal and oomycete colonization of plants. Current Biology, 22, 2242–2246. 10.1016/j.cub.2012.09.043 23122843

[fes3370-bib-0090] Wang, F. , Rose, T. , Jeong, K. , Kretzschmar, T. , & Wissuwa, M. (2016). The knowns and unknowns of phosphorus loading into grains, and implications for phosphorus efficiency in cropping systems. Journal of Experimental Botany, 67, 1221–1229. 10.1093/jxb/erv517 26662950

[fes3370-bib-0091] Wang, S. , Basten, C. J. , & Zeng, Z.‐B. (2012). Windows QTL Cartographer 2.5. Department of Statistics. North Carolina State University. http://statgen.ncsu.edu/qtlcart/WQTLCart.htm

[fes3370-bib-0092] Wang, S. , Wong, D. , Forrest, K. , Allen, A. , Chao, S. , Huang, B. E. , Maccaferri, M. , Salvi, S. , Milner, S. G. , Cattivelli, L. , Mastrangelo, A. M. , Whan, A. , Stephen, S. , Barker, G. , Wieseke, R. , Plieske, J. , International Wheat Genome Sequencing Consortium , Lillemo, M. , Mather, D. , … Akhunov, E. (2014). Characterization of polyploid wheat genomic diversity using a high‐density 90 000 single nucleotide polymorphism array. Plant Biotechnology Journal, 12, 787–796. 10.1111/pbi.12183 24646323 PMC4265271

[fes3370-bib-0093] Watts‐Williams, S. J. , Emmett, B. D. , Levesque‐Tremblay, V. , MacLean, A. M. , Sun, X. , Satterlee, J. W. , Fei, Z. , & Harrison, M. J. (2019). Diverse *Sorghum bicolor* accessions show marked variation in growth and transcriptional responses to arbuscular mycorrhizal fungi. Plant, Cell & Environment, 42, 1758–1774. 10.1111/pce.13509 30578745

[fes3370-bib-0094] White, P. J. , George, T. S. , Gregory, P. J. , Bengough, A. G. , Hallett, P. D. , & McKenzie, B. M. (2013). Matching roots to their environment. Annals of Botany, 112, 207–222. 10.1093/aob/mct123 23821619 PMC3698393

[fes3370-bib-0095] Withers, P. J. A. , & Haygarth, P. M. (2007). Agriculture, phosphorus and eutrophication: a European perspective. Soil Use and Management, 23, 1–4. 10.1111/j.1475-2743.2007.00116.x

[fes3370-bib-0096] Withers, P. J. A. , Hodgkinson, R. A. , Rollett, A. , Dyer, C. , Dils, R. , Collins, A. L. , Bilsborrow, P. E. , Bailey, G. , & Sylvester‐Bradley, R. (2017). Reducing soil phosphorus fertility brings potential long‐term environmental gains: A UK analysis. Environmental Research Letters, 12, 63001. 10.1088/1748-9326/aa69fc

[fes3370-bib-0097] Yang, H. , Zhang, Q. , Dai, Y. , Liu, Q. , Tang, J. , Bian, X. , & Chen, X. (2015). Effects of arbuscular mycorrhizal fungi on plant growth depend on root system: a meta‐analysis. Plant and Soil, 389, 361–374. 10.1007/s11104-014-2370-8

[fes3370-bib-0098] YEN (2021). YEN nutrition annual review. ADAS Crop Physiology Team.

[fes3370-bib-0099] Zeng, Z. B. (1994). Precision mapping of quantitative trait loci. Genetics, 136, 1457–1468. 10.1093/genetics/136.4.1457 8013918 PMC1205924

[fes3370-bib-0100] Zhang, S. , Lehmann, A. , Zheng, W. , You, Z. , & Rillig, M. C. (2019). Arbuscular mycorrhizal fungi increase grain yields: a meta‐analysis. New Phytologist, 222, 543–555. 10.1111/nph.15570 30372522

[fes3370-bib-0101] Zhu, Y. G. , Smith, S. E. , Barritt, A. R. , & Smith, F. A. (2001). Phosphorus (P) efficiencies and mycorrhizal responsiveness of old and modern wheat cultivars. Plant and Soil, 237, 249–255. 10.1023/A:1013343811110

[fes3370-bib-0102] Zipfel, C. , & Oldroyd, G. E. D. (2017). Plant signalling in symbiosis and immunity. Nature, 543, 328–336. 10.1038/nature22009 28300100

