# Supplementary material for: Variation in mycorrhizal growth response among a spring wheat mapping population shows potential to breed for symbiotic benefit
Source: Food Energy Secur. 2022 Feb 14;11(2):e370. doi: 10.1002/fes3.370 (PMC9286679; doi:10.1002/fes3.370)
Supplement: Supplementary file 1 — Appendix S1 [file FES3-11-0-s001.docx]

**Supporting Information Table S1.** Spring wheat lines from DH population of Avalon x Cadenza used for phenotype screen, from field material H2017.

| Population | Line | Population | Line | Population | Line |
| --- | --- | --- | --- | --- | --- |
| AxC DH | *AC1* | AxC DH | *AC80* | AxC DH | *AC134* |
| AxC DH | *AC2* | AxC DH | *AC81* | AxC DH | *AC135* |
| AxC DH | *AC5* | AxC DH | *AC84* | AxC DH | *AC137* |
| AxC DH | *AC6* | AxC DH | *AC85* | AxC DH | *AC138* |
| AxC DH | *AC7* | AxC DH | *AC87* | AxC DH | *AC139* |
| AxC DH | *AC8* | AxC DH | *AC88* | AxC DH | *AC140* |
| AxC DH | *AC16* | AxC DH | *AC92* | AxC DH | *AC141* |
| AxC DH | *AC17* | AxC DH | *AC93* | AxC DH | *AC143* |
| AxC DH | *AC18* | AxC DH | *AC96* | AxC DH | *AC145* |
| AxC DH | *AC19* | AxC DH | *AC98* | AxC DH | *AC146* |
| AxC DH | *AC21* | AxC DH | *AC99* | AxC DH | *AC147* |
| AxC DH | *AC22* | AxC DH | *AC100* | AxC DH | *AC148* |
| AxC DH | *AC23* | AxC DH | *AC101* | AxC DH | *AC150* |
| AxC DH | *AC24* | AxC DH | *AC102* | AxC DH | *AC152* |
| AxC DH | *AC27* | AxC DH | *AC103* | AxC DH | *AC156* |
| AxC DH | *AC28* | AxC DH | *AC104* | AxC DH | *AC158* |
| AxC DH | *AC41* | AxC DH | *AC105* | AxC DH | *AC162* |
| AxC DH | *AC42* | AxC DH | *AC107* | AxC DH | *AC163* |
| AxC DH | *AC43* | AxC DH | *AC108* | AxC DH | *AC164* |
| AxC DH | *AC44* | AxC DH | *AC109* | AxC DH | *AC165* |
| AxC DH | *AC46* | AxC DH | *AC114* | AxC DH | *AC166* |
| AxC DH | *AC52* | AxC DH | *AC115* | AxC DH | *AC167* |
| AxC DH | *AC53* | AxC DH | *AC116* | AxC DH | *AC168* |
| AxC DH | *AC54* | AxC DH | *AC117* | AxC DH | *AC170* |
| AxC DH | *AC55* | AxC DH | *AC119* | AxC DH | *AC172* |
| AxC DH | *AC58* | AxC DH | *AC120* | AxC DH | *AC173* |
| AxC DH | *AC62* | AxC DH | *AC122* | AxC DH | *AC176* |
| AxC DH | *AC65* | AxC DH | *AC125* | AxC DH | *AC180* |
| AxC DH | *AC70* | AxC DH | *AC126* | AxC DH | *AC186* |
| AxC DH | *AC71* | AxC DH | *AC129* | AxC DH | *AC188* |
| AxC DH | *AC73* | AxC DH | *AC130* | AxC DH | *AC191* |
| AxC DH | *AC74* | AxC DH | *AC132* | AxC DH | *AC192* |
| AxC DH | *AC79* | AxC DH | *AC133* | AxC DH | *Cadenza* |

**Supporting Information Table S2.** Planting and harvesting timings for experimental blocks 1-5. Each block contained one replicate pair (mycorrhizal and non-mycorrhizal) from the 99 spring lines selected from the Avalon x Cadenza double haploid mapping population.

| Block | Planted | Harvested |
| --- | --- | --- |
| 1 | 11th – 12^th^ July | 15^th^ – 17^th^ August |
| 2 | 18^th^ – 19^th^ July | 22^nd^ – 24^th^ August |
| 3 | 25^th^ – 26^th^ July | 29^th^ – 31^st^ August |
| 4 | 1^st^ – 2^nd^ August | 5^th^ – 7^th^ September |
| 5 | 8^th^ – 9^th^ August | 12^th^ – 14^th^ September |

**Supporting information Table S3.** Protocol for amended Long Ashton nutrient solution preparation.

| Compound | Compound formula | g/L in prepared solution |
| --- | --- | --- |
| *Macronutrients* | | |
| Calcium nitrate | Ca(NO_3_)_2_ | 0.6420 |
| Potassium nitrate | KNO_3_ | 0.4048 |
| Magnesium sulfate heptahydrate | MgSO_4_. 7H_2_O | 0.3680 |
| Monosodium phosphate dihydrate | NaH_2_PO_4_.2H_2_O | 0.0520 |
| *Micronutrients* | | |
| Sodium ferric EDTA | NaFeEDTA | 0.03350 |
| Sodium chloride | NaCl | 0.00585 |
| Boric acid | H_3_BO_3_ | 0.00310 |
| Manganese sulfate tetrahydrate | MnSO_4_.4H_2_O | 0.00223 |
| Zinc sulfate heptahydrate | ZnSO_4_.7H_2_O | 0.00029 |
| Copper sulfate pentahydrate | CuSO_4_.5H_2_O | 0.00025 |
| Sodium molybdate dihydrate | NaMoO_4_.2H_2_O | 0.00012 |

**Supporting Information Table S4.** Replicates excluded from analysis - plants which did not grow are listed, shown separately for the mycorrhizal and non-mycorrhizal groups.

| Block | Mycorrhizal | Non-mycorrhizal |
| --- | --- | --- |
| 1 | 18, 114, 147 | 8, 138, Cadenza |
| 2 | 80, 119, 130 | 24, 27, 92, 167, 170 |
| 3 | 5, 6, 18, 19, 21, 23, 28, 53, 58, 87, 114, 129, 130, 152, 176, 191 | 5, 7, 23, 42, 58, 71, 74, 81, 96, 98, 100, 114, 122, 135, 138, 180 |
| 4 | 8, 101, 105 | 147 |
| 5 | 43, 119, 132, 147 | 84, 108, 115, 117 |

**Supporting Information Table S5.** List of wheat (*Triticum aestivum* L.) lines taken from the Avalon x Cadenza DH mapping population, used for quantification of phosphorus content and concentration in aboveground tissue, and root colonisation.

| Lines used | | | | |
| --- | --- | --- | --- | --- |
| Phosphorus assay | | | AMF colonisation | |
| 1 | 85 | 139 | 7 | 122 |
| 5 | 88 | 141 | 8 | 129 |
| 7 | 93 | 145 | 16 | 132 |
| 16 | 98 | 148 | 21 | 134 |
| 18 | 100 | 152 | 27 | 135 |
| 21 | 102 | 158 | 41 | 138 |
| 23 | 104 | 163 | 44 | 140 |
| 27 | 107 | 165 | 52 | 141 |
| 41 | 109 | 167 | 55 | 146 |
| 43 | 116 | 170 | 80 | 147 |
| 52 | 120 | 173 | 85 | 152 |
| 53 | 122 | 180 | 93 | 156 |
| 55 | 125 | 188 | 98 | 165 |
| 62 | 126 | 192 | 100 | 166 |
| 70 | 130 |  | 103 | 167 |
| 73 | 133 |  | 104 | 186 |
| 79 | 135 |  | 105 | 188 |
| 81 | 137 |  | 117 | Cadenza |

**Supporting Information Table S6.** Categorisation of lines as Negative, Neutral and Positive responders, sorted by mean shoot dry biomass response to inoculation with mix of 5 arbuscular mycorrhizal fungal species.

| Line | MGR | MGR response group | Root length colonised | Arbuscule frequency | Vesicle frequency |
| --- | --- | --- | --- | --- | --- |
| 132 | -33.90 | Negative | 44.92 | 8.32 | 1.75 |
| 103 | -32.26 | Negative | 44.80 | 3.40 | 1.20 |
| 52 | -28.57 | Negative | 44.63 | 3.12 | 0.20 |
| 186 | -24.75 | Negative | 38.00 | 4.33 | 3.00 |
| 7 | -16.25 | Negative | 37.00 | 8.33 | 1.67 |
| 8 | -15.69 | Negative | 45.72 | 5.62 | 1.33 |
| 98 | -14.74 | Negative | 33.67 | 3.67 | 1.00 |
| 105 | -11.29 | Negative | 30.00 | 1.33 | 0.00 |
| 167 | -8.92 | Negative | 53.00 | 7.00 | 2.00 |
| 55 | -8.54 | Negative | 40.50 | 9.76 | 3.00 |
| 134 | -4.76 | Negative | 36.67 | 3.00 | 0.33 |
| 156 | -4.29 | Negative | 45.67 | 5.00 | 1.00 |
| 104 | -1.61 | Neutral | 32.00 | 5.00 | 0.00 |
| 16 | 2.82 | Neutral | 23.33 | 2.67 | 0.67 |
| 135 | 5.76 | Neutral | 39.33 | 4.33 | 1.33 |
| 100 | 10.00 | Neutral | 48.43 | 9.57 | 1.18 |
| 44 | 11.48 | Neutral | 51.33 | 10.67 | 2.00 |
| 140 | 12.50 | Neutral | 33.33 | 3.00 | 0.67 |
| 93 | 12.79 | Neutral | 34.33 | 11.00 | 2.33 |
| 129 | 13.21 | Neutral | 34.67 | 5.00 | 0.00 |
| 27 | 15.56 | Neutral | 52.22 | 7.67 | 3.00 |
| 122 | 16.92 | Neutral | 47.00 | 5.33 | 1.33 |
| 117 | 21.54 | Neutral | 37.38 | 4.43 | 1.00 |
| 165 | 24.64 | Neutral | 46.67 | 9.00 | 0.33 |
| 41 | 32.43 | Positive | 21.67 | 1.67 | 0.33 |
| 138 | 35.48 | Positive | 39.33 | 6.67 | 0.67 |
| 152 | 35.64 | Positive | 36.67 | 5.67 | 1.33 |
| 166 | 35.80 | Positive | 25.67 | 4.67 | 0.33 |
| 147 | 37.14 | Positive | 25.26 | 0.64 | 0.00 |
| 146 | 44.19 | Positive | 44.93 | 10.45 | 2.43 |
| Cadenza | 54.17 | Positive | 32.33 | 3.00 | 1.33 |
| 188 | 55.00 | Positive | 44.57 | 10.85 | 2.83 |
| 85 | 58.49 | Positive | 16.67 | 1.33 | 0.33 |
| 80 | 66.67 | Positive | 31.69 | 7.58 | 2.75 |
| 141 | 73.44 | Positive | 41.99 | 6.98 | 1.59 |
| 21 | 89.02 | Positive | 45.20 | 7.33 | 3.70 |

**Supporting Information Table S7.** Wilcoxon signed rank exact test outputs for mycorrhizal growth response (MGR) traits. Lines shown are those where tests indicated that line MGR was significantly greater than zero. Where test indicated line MGR was not significantly different from zero, cells are assigned the symbol “–”.

| Mycorrhizal growth response significantly ***greater*** than zero | | | | | | | | | | |
| --- | --- | --- | --- | --- | --- | --- | --- | --- | --- | --- |
|  | **Shoot dry biomass** | | **Root dry biomass** | | **Total dry biomass** | | **Root weight ratio** | | **Shoot height** | |
| **Line** | V | *p* | V | *p* | V | *p* | V | *p* | V | *p* |
| 19 | – | – | – | – | – | – | – | – | **10** | **0.049** |
| 21 | **10** | **0.049** | – | – | – | – | – | – | – | – |
| 70 | – | – | – | – | – | – | **15** | **0.031** | – | – |
| 74 | **15** | **0.029** | – | – | **15** | **0.031** | – | – | – | – |
| 81 | – | – | – | – | – | – | – | – | **15** | **0.029** |
| 84 | – | – | – | – | – | – | – | – | **15** | **0.031** |
| 85 | **15** | **0.031** | – | – | – | – | – | – | – | – |
| 87 | – | – | – | – | – | – | – | – | **10** | **0.049** |
| 88 | – | – | – | – | – | – | **15** | **0.031** | **15** | **0.031** |
| 96 | **15** | **0.029** | – | – | – | – | – | – | **15** | **0.031** |
| 107 | – | – | – | – | – | – | – | – | **15** | **0.031** |
| 117 | – | – | **15** | **0.031** | – | – | **15** | **0.031** | – | – |
| 129 | – | – | – | – | – | – | – | – | **10** | **0.049** |
| 139 | – | – | – | – | – | – | – | – | **15** | **0.029** |
| 146 | **15** | **0.029** | – | – | – | – | – | – | **15** | **0.029** |
| 148 | – | – | – | – | – | – | – | – | **15** | **0.031** |
| 166 | **15** | **0.029** | – | – | **15** | **0.031** | – | – | – | – |
| 172 | – | – | **15** | **0.031** | **15** | **0.031** | – | – | – | – |
| 176 | **10** | **0.049** | – | – | – | – | – | – | – | – |
| 188 | **15** | **0.029** | **15** | **0.031** | **15** | **0.031** | – | – | **15** | **0.029** |
| 192 | – | – | – | – | – | – | – | – | **15** | **0.031** |
| Cadenza | **15** | **0.031** | **15** | **0.031** | **15** | **0.031** | – | – | **15** | **0.031** |

| Mycorrhizal growth response significantly ***lower*** than zero | | | | | | | | | | |
| --- | --- | --- | --- | --- | --- | --- | --- | --- | --- | --- |
|  | **Shoot dry biomass** | | **Root dry biomass** | | **Total dry biomass** | | **Root weight ratio** | | **Shoot height** | |
| **Line** | V | *p* | V | *p* | V | *p* | V | *p* | V | *p* |
| 42 | – | – | – | – | – | – | **0** | **0.031** | – | – |
| 52 | **0** | **0.029** | **0** | **0.031** | **0** | **0.031** | – | – | – | – |
| 54 | – | – | **0** | **0.031** | – | – | **0** | **0.031** | – | – |
| 84 | – | – | – | – | – | – | **0** | **0.031** | – | – |
| 92 | – | – | **0** | **0.031** | **0** | **0.031** | – | – | – | – |
| 98 | – | – | – | – | – | – | **0** | **0.031** | – | – |
| 99 | – | – | **0** | **0.031** | – | – | **0** | **0.031** | – | – |
| 103 | – | – | **0** | **0.031** | **0** | **0.031** | – | – | – | – |
| 115 | – | – | – | – | – | – | **0** | **0.031** | – | – |
| 116 | – | – | **0** | **0.031** | **0** | **0.031** | – | – | **0** | **0.029** |
| 126 | – | – | – | – | – | – | **0** | **0.031** | – | – |
| 132 | **0** | **0.049** | – | – | – | – | – | – | – | – |
| 180 | – | – | **0** | **0.031** | **0** | **0.031** | **0** | **0.031** | – | – |
| 186 | **0** | **0.029** | **0** | **0.031** | **0** | **0.031** | – | – | – | – |

**Supporting Information Table S8.** Wilcoxon signed rank exact test outputs for mycorrhizal growth response (MGR) traits. Lines shown are those where tests indicated that line MGR was significantly lower than zero. Where test indicated line MGR was not significantly different from zero, cells are assigned the symbol “–”.

**Supporting Information Figure S1.** Comparison of (a) total dry biomass (b) root weight ratio and (c) shoot height in mycorrhiza-inoculated and mock-inoculated wheat (*Triticum aestivum* L.) plants. Boxes sharing letters are not significantly different, as determined by Wilcoxon signed rank test. Blue diamonds represent mean values for boxplot data. Root weight ratio represents root dry biomass as a proportion of total dry biomass. All replicate plants of 99 lines of Avalon x Cadenza DH mapping population are represented, n = 445.

**Supporting Information Figure S2***.* Response of wheat (*Triticum aestivum L*.) root dry biomass to arbuscular mycorrhizal inoculation. Boxes represent individual wheat lines from the Avalon x Cadenza DH mapping population. Wheat lines are ranked by mean response to inoculation. Blue diamonds on boxes represent mean MGR value for that line. Grey stars on the x-axis denote lines where Wilcoxon signed rank test show the mean is significantly lower than zero; yellow stars show lines where mean MGR value is significantly higher than zero. Except where noted in Supporting Information Table 3, n = 5.

**Supporting Information Figure S3.** Response of wheat (*Triticum aestivum* L.) total dry biomass to arbuscular mycorrhizal inoculation. Boxes represent individual lines from the Avalon x Cadenza DH mapping population. Wheat lines are ranked by mean response to inoculation. Blue diamonds on boxes represent mean MGR value for that line. Grey stars on the x-axis denote lines where Wilcoxon signed rank test show the mean is significantly lower than zero; yellow stars show lines where mean MGR value is significantly higher than zero. Except where noted in Supporting Information Table 3, n = 5.

**Supporting Information Figure S4***.* Response of wheat (*Triticum aestivum* L.) root weight ratio to arbuscular mycorrhizal inoculation. Boxes represent individual wheat lines from the Avalon x Cadenza DH mapping population. Wheat lines are ranked by mean response to inoculation. Blue diamonds on boxes represent mean MGR value for that line. Grey stars on the x-axis denote lines where Wilcoxon signed rank test show the mean is significantly lower than zero; yellow stars show lines where mean MGR value is significantly higher than zero. Root weight ratio represents root dry biomass as a proportion of total dry biomass. Except where noted in Supporting Information Table 3, n = 5.

**Supporting Information Figure S5***.* Response of wheat (*Triticum aestivum* L.) shoot height to arbuscular mycorrhizal inoculation. Boxes represent individual wheat lines from the Avalon x Cadenza DH mapping population. Wheat lines are ranked by mean response to inoculation. Blue diamonds on boxes represent mean MGR value for that line. Grey stars on the x-axis denote lines where Wilcoxon signed rank test show the mean is significantly lower than zero; yellow stars show lines where mean MGR value is significantly higher than zero. Except where noted in Supporting Information Table 3, n = 5.

**

**Supporting Information Figure S6***.* Scatterplot of wheat (*Triticum aestivum* L.) root dry biomass and shoot phosphorus concentration, with mycorrhizal-inoculated and mock-inoculated plotted separately in pink and blue, respectively. Statistical output shows results of Spearman rank correlation. Root dry biomass data are means of 3-5 replicates from each line; phosphorus data represent single replicates from 50 lines of Avalon x Cadenza DH mapping population are represented, n = 50.

**Supporting Information Figure S7***.* Comparison of root length colonisation in lines displaying contrasting shoot dry biomass response to mycorrhizal inoculation (negative, neutral, and positive). Mean values for 3-5 replicate plants of 12 lines (lines were selected for their response to inoculation) are represented in each MGR group. Assignment of lines to MGR groups is detailed in Supporting Information Table 6.

**Supporting Information Figure S8.** Spearman rank correlation plots of mycorrhizal colonisation traits against wheat *(Triticum aestivum L.)* growth and nutrition traits: a) shoot dry biomass response to AMF inoculation plotted against AMF vesicle frequency; (b) shoot P concentration in AM-inoculated plants, plotted against AMF vesicle frequency; shoot P content plotted against (c) root length colonized by AMF, (d) arbuscule frequency and (e) vesicle frequency. Colonisation data points represent means of plant trait data from 3-5 replicates. Phosphorus data points represent individual plant values from selected lines (lines used are listed in Supporting Information Table 5).

**

**Supporting Information Figure S9.**  Spearman rank correlation plots of mycorrhizal colonisation traits against wheat *(Triticum aestivum L.)* growth and nutrition traits: AM plant root dry biomass plotted against (a) root length colonized; (b) arbuscule frequency and (c) vesicle frequency. AM shoot dry biomass plotted against (d) root length colonised, (e) arbuscule frequency and (f) vesicle frequency. Colonisation data points represent means of plant trait data from 3-5 replicates of selected lines (lines used are listed in Supporting Information Table 5).

**Supporting Information Figure S10.** Spearman rank correlation plots of mycorrhizal colonisation traits against wheat *(Triticum aestivum L.)* growth and nutrition traits. Effect of (a) NM shoot P concentration and (b) NM shoot P content on root length colonisation; effect of (c) NM shoot P concentration and (d) NM shoot P content on arbuscule frequency. Association between (e) NM shoot P concentration, (f) NM shoot P content and shoot P response to inoculation in terms of shoot P concentration (MPR concentration) and content (MPR content), respectively. Association between shoot mycorrhizal growth response (MGR) and (g) NM shoot P concentration and (h) NM shoot P content. Colonisation data are means of 3-5 replicates of selected lines, while phosphorus data are from individual plants (lines used are listed in Supporting Information Table 5).

**Supporting Information Figure S11.** Spearman rank correlation plots of mycorrhizal shoot biomass response to inoculation against wheat *(Triticum aestivum L.)* shoot phosphorus response to inoculation. Shoot biomass response to inoculation plotted against (a) shoot phosphorus concentration response to inoculation and (b) shoot phosphorus content response to inoculation.
